# Optimized Design of Low-Carbon Fly Ash–Slag Composite Concrete Considering Carbonation Durability and CO_2_ Concentration Rising Impacts

**DOI:** 10.3390/ma18143418

**Published:** 2025-07-21

**Authors:** Kang-Jia Wang, Seung-Jun Kwon, Xiao-Yong Wang

**Affiliations:** 1 Department of Integrated Energy and Infra System, Kangwon National University, Chuncheon-si 24341, Republic of Korea; wangkangjia@kangwon.ac.kr; 2 Department of Civil and Environmental Engineering, Hannam University, Daedeok-gu, Daejeon 34430, Republic of Korea; 3 Department of Architectural Engineering, Kangwon National University, Chuncheon-si 24341, Republic of Korea

**Keywords:** low-carbon concrete, carbonation durability, CO_2_ concentration rising, fly ash, slag, genetic algorithm

## Abstract

Fly ash and slag are widely used as mineral admixtures to partially replace cement in low-carbon concrete. However, such composite concretes often exhibit a greater carbonation depth than plain Portland concrete with the same 28-day strength, increasing the risk of steel reinforcement corrosion. Previous mix design methods have overlooked this issue. This study proposes an optimized design method for fly ash–slag composite concrete, considering carbonation exposure classes and CO_2_ concentrations. Four exposure classes are addressed—XC1 (completely dry or permanently wet environments such as indoor floors or submerged concrete), XC2 (wet but rarely dry, e.g., inside water tanks), XC3 (moderate humidity, e.g., sheltered outdoor environments), and XC4 (cyclic wet and dry, e.g., bridge decks and exterior walls exposed to rain). Two CO_2_ levels—0.04% (ambient) and 0.05% (elevated)—were also considered. In Scenario 1 (no durability constraint), the optimized designs for all exposure classes were identical, with 60% slag and 75% total fly ash–slag replacement. In Scenario 2 (0.04% CO_2_ with durability), the designs for XC1 and XC2 remained the same, but for XC3 and XC4, the carbonation depth became the controlling factor, requiring a higher binder content and leading to compressive strengths exceeding the target. In Scenario 3 (0.05% CO_2_), despite the increased carbonation depth, the XC1 and XC2 designs were unchanged. However, XC3 and XC4 required further increases in binder content and actual strength to meet durability limits. Overall, compressive strength governs the design for XC1 and XC2, while carbonation durability is critical for XC3 and XC4. Increasing the water-to-binder ratio reduces strength, while higher-strength mixes emit more CO_2_ per cubic meter, confirming the proposed method’s engineering validity.

## 1. Introduction

Concrete is the most widely used construction material globally, being extensively applied in roads, bridges, and building structures [1,2,3]. However, traditional cement production emits significant amounts of carbon dioxide, accounting for over 7% of the global carbon emissions [4]. With the intensification of global warming, the green transformation of the construction industry is imperative. Developing and promoting low-carbon concrete can effectively reduce greenhouse gas emissions and contribute to environmental sustainability throughout the lifecycle of buildings.

The simplest way to cut carbon emissions is by substituting part of the cement with fly ash or slag [5,6,7,8,9]. Currently, many researchers have proposed material design methods for concrete that incorporate admixtures. These design methods can be roughly divided into two categories. The first category is similar to the traditional concrete design method, while the second category involves the application of machine learning methods.

The first type of design method establishes the relationship between various properties of concrete (such as strength, workability, and carbon emissions) and its material composition (such as the water–binder mass ratio, mineral admixture replacement rate, water content per unit volume, etc.), before obtaining the mix ratio based on an analytical method. The main representative works of the first type of design methods are as follows. In 2015, Yang et al. [10] studied supplementary cementitious materials (SCMs) to reduce CO_2_ emissions in concrete production, using 9209 mix data to formulate equations for binder and CO_2_ intensities, demonstrating that GGBS significantly lowers emissions while meeting strength targets. In 2016, Miller et al. [11] created equations to predict the compressive strength and global warming potential (GWP) of fly ash and GGBS concrete, optimizing the water-to-binder ratios to minimize the GWP per strength unit; they found that a high GGBS content was sustainable for high-strength mixes, though cement-only mixes may suit lower strengths. Esmaeilkhanian et al. (2017) [12] developed a mix design approach for Eco-SCC that limited the powder content to 315 kg/m^3^ and optimized the sand-to-coarse aggregate ratio based on the Funk and Dinger particle packing model and powder composition via rheological measurements, achieving satisfactory workability, a 25–35 MPa 28-day compressive strength, and low CO_2_ emissions (170–232 kg/m^3^) for commercial and housing applications. In 2018, Jiao et al. [13] proposed a simplex centroid design to optimize concrete mix proportions, achieving flowability (slump: 160–230 mm), rheological properties (yield stress: 110–290 Pa; viscosity: 12–25 Pa·s), and strength (40–47 MPa at 28 days) for ternary paste–aggregate and cement–fly ash–slag systems. This work was validated experimentally but was limited by empirical workability metrics. In 2020, Li and colleagues [14] studied an advanced SCC mix design using fly ash, based on paste rheology thresholds and packing density characteristics, achieving prediction accuracies of over 83%, compared to the initial method’s maximum of 60%.

The main feature of the second type of design method is to use machine learning methods to determine the relationship between the various properties of concrete and the material composition, before obtaining the concrete mix ratio through heuristic algorithms. The main representative works of the second type of design methods are as follows. Golafshani and Behnood (2019) [15] employed biogeography-based programming (BBP) to model the strength of silica fume concrete and applied constrained biogeography-based optimization (CBBO) to determine the optimal mix proportions, achieving high accuracy (Pearson’s correlation: 0.93) and cost-effective 40–100 MPa mixes with a 1030-record dataset and user-friendly GUI. In 2019, Kurda et al. [16] introduced CONCRETop, a multi-criteria decision method for optimizing concrete mixes, balancing technical performance, cost, and environmental impact; it was validated through case studies for various applications. In 2020, Zhang et al. [17] employed machine learning (BPNN for UCS; RF for slump) and multi-objective particle swarm optimization (MOPSO) to optimize concrete mixes, delivering Pareto-optimal solutions for high-performance and plastic concrete, reducing costs while meeting strength and workability needs. In 2022, Bhuva and Bhogayata [18] reviewed artificial neural networks (ANNs) for self-compacting concrete mix design, achieving a high accuracy in predicting strength and rheological properties; this was validated experimentally, although automating conventional testing remains challenging. In 2023, Shobeiri et al. [19] optimized concrete with fly ash and slag using AI, genetic algorithms, life-cycle assessment, and economic modeling, showing that chemical composition, cement content, and binder type significantly affect GWP and cost, with optimized mixes reducing both emissions and costs for high-strength concrete. In 2024, Gao et al. [20] proposed a framework using a Variational Autoencoder (VAE), XGBoost, and NSGA-II to design sustainable concrete, reducing the design space from 7D to 2D, subsequently optimizing cost, CO_2_ emissions, and strength. This was validated on the UCI dataset with transparent SHAP explanations. In 2024, Le Nguyen et al. [21] introduced a generative AI framework with machine learning (XGBoost, CatBoost, and LGBM) and NSGA-II to design concrete, balancing strength, cost, and CO_2_; this was validated experimentally.

Although previous studies have extensively investigated low-carbon concrete with admixtures [10,11,12,13,14,15,16,17,18,19,20,21], they have several shortcomings. Firstly, prior mix design research primarily focused on strength, workability, cost, and carbon emissions, neglecting carbonation durability constraints [10,11,12,13,14,15,16,17,18,19,20,21]. For concrete containing fly ash and slag, when achieving the same 28-day strength as Portland concrete, it exhibits a higher carbonation depth, increasing the risk of steel reinforcement corrosion due to carbonation [22]. Secondly, according to durability design codes, carbonation durability exposure classes are classified into four levels from mild to severe, as follows: XC1 (fully saturated or completely dry), XC2 (high humidity with occasional drying), XC3 (moderate relative humidity), and XC4 (wet–dry cycles) [23]. Whether all four carbonation exposure classes (XC1, XC2, XC3, and XC4) need to consider carbonation effects in mix design optimization requires further investigation. Lastly, in relation to fossil fuel combustion, industrial processes, and deforestation, rising CO_2_ concentrations in the atmosphere accelerate carbonation depth growth. Under the combined influence of increasing CO_2_ concentrations and more-severe carbonation durability exposure classes (XC3 and XC4), further research relating to the methods with which to meet carbonation durability requirements through mix design is also warranted.

To overcome the limitations of earlier research, this study presents a design approach for low-carbon slag–fly ash concrete that incorporates carbonation exposure conditions and the effects of increasing CO_2_ concentrations. This study sets the reduction in per-cubic-meter carbon emissions of concrete as the optimization objective, taking into account strength, workability, admixture replacement rates, and carbonation durability. Through three design scenarios (without considering carbonation durability, considering carbonation durability at 0.04% CO_2_ concentration, and at 0.05% CO_2_ concentration) combined with four carbonation durability exposure classes (XC1, XC2, XC3, and XC4), twelve optimized mix designs were obtained using a genetic algorithm that considers the target function and constraints (three design scenarios × four carbonation durability exposure classes = twelve optimized mix designs). The proposed design method clarifies the determining factors for mix design (strength-driven or carbonation durability-driven), provides an approach to address CO_2_ concentration increases through mix design, and is of great engineering relevance to the design of low-carbon fly ash–slag composite concrete.

Compared with the analytical and machine learning methods in previous studies, the unique contributions of this paper are as follows: First, in this study, the constraint of carbonation durability is considered when optimizing the design of low-carbon concrete. Previous researchers did not consider the constraint of carbonation durability when optimizing the design of low-carbon concrete. Using the methods of previous researchers may lead to insufficient carbonation durability and cause steel corrosion. Second, this paper distinguishes different control factors in the design process of low-carbon concrete, such as compressive strength control or carbonation durability control. In the methods used by previous researchers, compressive strength is simply considered to control the mixture design of low-carbon concrete. Third, as the CO_2_ concentration increases, the carbonation depth of concrete increases, and the mixture design needs to be adjusted to ensure sufficient carbonation durability. This paper provides a specific mixture design method that considers the increase in CO_2_ concentration. The design methods proposed by previous researchers did not provide a similar material design method for low-carbon concrete to resist rising CO_2_ concentrations.

## 2. Mixed Design Method

**Figure 1:**
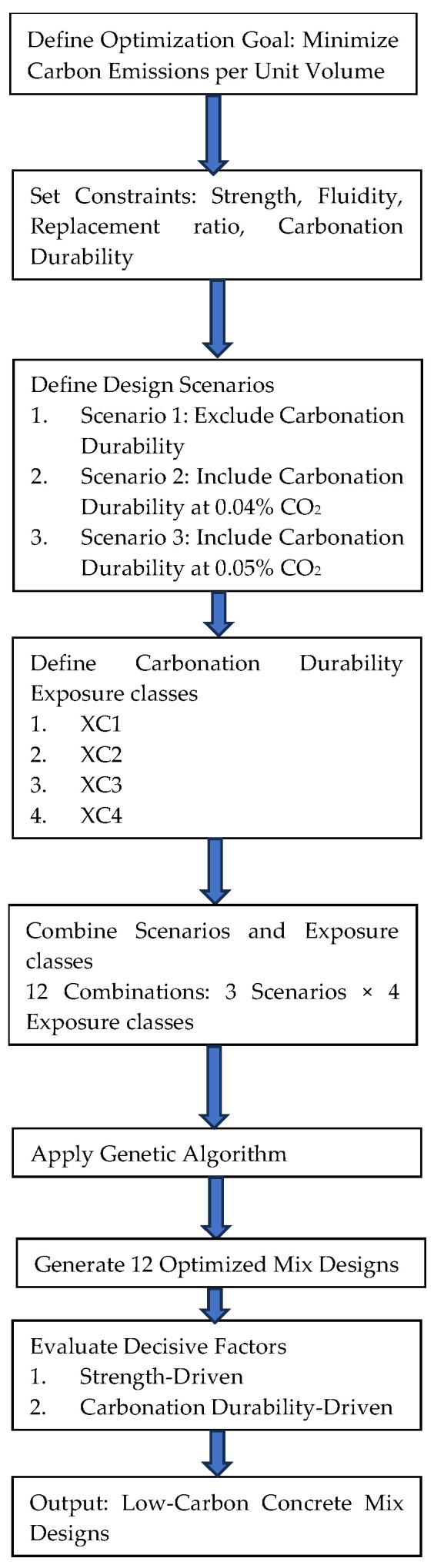
Flowchart of proposed model.

A schematic of the proposed method is illustrated in Figure 1. The flowchart outlines a systematic process for optimizing concrete mix designs to minimize carbon emissions per unit volume. The process begins by defining the optimization goal, followed by setting constraints including strength, fluidity, replacement ratio, and carbonation durability. It then involves defining three design scenarios—excluding carbonation durability, including it at 0.04% CO_2_, and including it at 0.05% CO_2_. Next, carbonation durability exposure classes (XC1, XC2, XC3, and XC4) are identified, leading to 12 unique combinations by pairing the scenarios with the exposure classes. A genetic algorithm is applied to generate 12 optimized mix designs, which are subsequently evaluated based on strength-driven and carbonation durability-driven factors. The process concludes by outputting low-carbon concrete mix designs that balance the optimization goal with the established constraints, tailored to various environmental conditions and performance requirements.

### 2.1. Carbon Emission Calculation Model

In cement-based materials, the main source of carbon emissions in concrete is the binder components, including cement, fly ash, and slag. Although other components, such as aggregates and chemical admixtures, also emit CO_2_, their contribution is relatively small compared to that of binders [10]. The equation for calculating the per-cubic-meter carbon emissions of concrete, *CO*_2*V*_, is as follows:

*CO*_2*V*_ = 0.93 × *CE* + 0.0196 × *FA* + 0.0265 × *SG* (1)

where *CE*, *FA*, and *SG* represent the masses of cement, fly ash, and slag per unit volume of concrete, respectively, and 0.93, 0.0196, and 0.0265 represent the CO_2_ emissions per unit mass of cement, fly ash, and slag, respectively [10]. The carbon emission calculation part only considers the emissions of the materials themselves, ignoring the emissions from transportation and production processes. If the emissions from transportation and production processes are taken into account, the carbon emissions per kilogram of cement, slag, and fly ash will increase, but the percentage of increase is different. The increase in cement is relatively small, while the increase in slag and fly ash is larger. This is because the carbon dioxide emitted during the production of a unit mass of cement is much higher than that of slag and fly ash.

### 2.2. Constraints for Optimization Design

Based on research by Yeh [24,25], when using machine learning algorithms for the optimization design of low-carbon concrete mix proportions, the optimization objective is the carbon emissions of the concrete. The constraints for the optimization design include compressive strength, workability, water/binder mass ratio, and the replacement rate of mineral admixtures [26]. In addition to these common constraints, the constraint of carbonation durability must also be considered. The methods for considering each constraint are as follows.

#### 2.2.1. Compressive Strength Constraint

To meet compressive strength requirements, the concrete must reach a strength greater than the standard-specified value at a certain age, usually 28 days. Design codes set the minimum 28-day compressive strength at 30 MPa [23,27]. This is expressed as follows:

*FC*28 > 30 MPa (2)

*FC*28 represents the real compressive strength achieved at 28 days.

For cement-based materials with a ternary system of cement, slag, and fly ash, the 28-day strength can be predicted using the following equation [28]:

*FC*28 = *A*1 × *CE*/*WT* + *A*2 × *SG*/*WT* + *A*3 × *FA*/*WT* + *A*4 (3)

In Equation (3), *WT* denotes the mass of water per cubic meter of concrete; *A*1, *A*2, and *A*3 are the strength coefficients for cement, slag, and fly ash, respectively; and *A*4 is the constant term in the strength prediction equation.

Yeh collected compressive strength design mix data from various researchers [24,25,26], comprising a total of 425 experimental results for 28-day compressive strength with different water-to-binder ratios and mineral admixture replacement rates. These experimental results are available online [29]. Based on the experimental results from the data collected by Yeh, the specific form of the 28-day strength prediction equation is as follows:

*FC*28 = 21.761 × *CE/WT* + 16.878 × *SG/WT* + 8.944 × *FA/WT* + (−6.51) (4)

**Figure 2:**
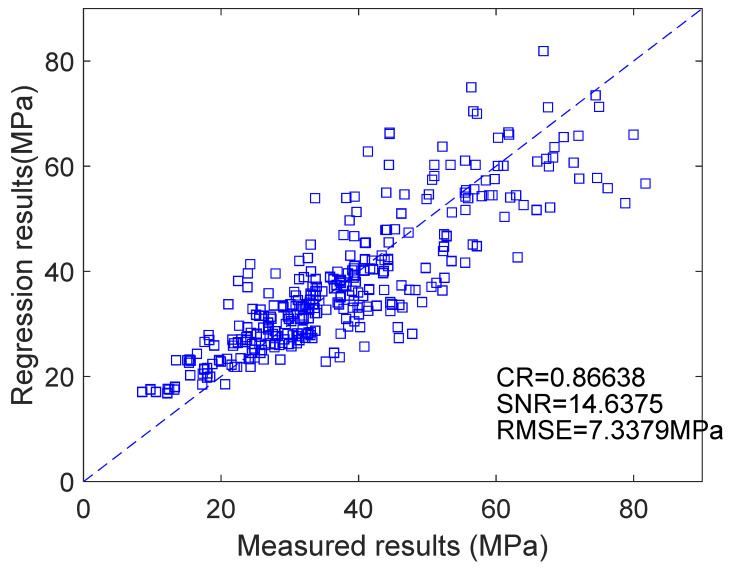
Comparison of design strength and predicted strength.

As shown in Figure 2, the predicted compressive strength closely matches the experimental results, with a correlation coefficient of 0.86638, a root mean square error (RMSE) of 7.3379 MPa, and a signal-to-noise ratio of 14.6375. These errors likely stem from the use of data from different researchers, which introduces variability due to differences in raw materials, concrete mixing methods, and curing conditions, leading to discrepancies between the estimated and measured results. Although the form of the strength Equation (4) is simple, its prediction accuracy is relatively high, offering a certain engineering reference value.

#### 2.2.2. Workability Constraint

The workability constraint is addressed through the unit water content. Based on previous studies, for commonly used engineering concrete with a maximum coarse aggregate size of 20 mm, a unit water content of 170 kg per cubic meter ensures that the concrete’s workability meets the requirements [30]. It should be noted that concrete workability is a highly complex issue. Using a constant unit water content is a simplified approach, and future research should further adopt models based on workability mechanisms to estimate concrete workability.

#### 2.2.3. Replacement Rate Constraint

Based on the experimental data collected by Yeh [24,25,26,29], the constraints for replacement rates are as follows. The proportions of fly ash, slag, and total mineral admixtures in the binder, represented by *FA*/(*CE + FA + SG*), *SG*/(*CE + FA + SG*), and (*FA + SG*)/(*CE + FA + SG*), must not exceed 0.55, 0.6, and 0.7, respectively [24,25,26,29].

#### 2.2.4. Carbonation Durability Constraint

For low-carbon concrete containing slag and fly ash, when its compressive strength is the same as that of Portland cement concrete, the low-carbon concrete containing slag and fly ash has a stronger resistance to chloride ion corrosion. When the compressive strength meets the requirements, the durability against chloride ion corrosion will also meet the requirements, and there is no need to consider the durability against chloride ion corrosion separately. As such, this study does not consider chloride ingression durability and only considers carbonation durability.

The carbonation durability constraint stipulates that the carbonation depth must remain less than the concrete cover depth, protecting steel reinforcement throughout the structure’s service life, which is expressed as follows [31]:

*Xc*(*t*) <= *CD* (5)

Here, *Xc* stands for carbonation depth, t indicates the carbonation duration, and *CD* represents the concrete cover depth over the reinforcement. According to concrete durability design codes, for carbonation durability, the value of *CD* is typically 25 mm [23,27].

According to durability codes, carbonation exposure classes are classified into four types according to relative humidity—XC1 (permanently wet or permanently dry), XC2 (wet with occasional drying), XC3 (moderate humidity), and XC4 (cyclic wet–dry conditions) [23].

According to research by Papadakis et al. [31,32,33], the equation for calculating carbonation depth is as follows:

(6) $$X_{C}=RWT\times \sqrt{\frac{2D{\left[CO_{2}\right]}_{0}t}{0.218\times \alpha_{H}\times (CE+0.5\times FA+0.7\times SG)}}$$

(7) $$\begin{array}{l} D=6.1\times 10^{-6}{\left(\frac{\left[WT-0.267\times \alpha_{H}\times (CE+0.5\times FA+0.7\times SG)\right]/1000}{\frac{CE+0.5\times FA+0.7\times SG}{\rho_{c}}+\frac{WT}{\rho_{w}}}\right)}^{3}\\ \;\;\;\times {\left(1-\frac{RH}{100}\right)}^{2.2}\times \exp\left[\beta (\frac{1}{T_{ref}}-\frac{1}{T})\right] \end{array}$$

In Equation (6), *RWT* represents the influence coefficient of wet–dry cycles on carbonation depth [27,34]. For the XC4 environmental condition, based on investigations of carbonation depth under wet–dry cycle conditions, the value of *RWT* ranges between 1.5 and 2. For concrete with mineral admixtures, the use of mineral admixtures fills the interfacial zone between the aggregate and the paste, so *RWT* is recommended to take the lower boundary value of 1.5 [34,35]. For XC1, XC2, and XC3, as there is no influence from wet–dry cycles, *RWT* is equal to 1.0 [27,34].

In Equation (6), *D* in the numerator represents the CO_2_ diffusion coefficient, [*CO*_2_]_0_ represents the ambient CO_2_ concentration, and $\alpha_{H}$ in the denominator represents the average reaction degree of the binder materials, where $\alpha_{H}=1-\exp(-3.38\times \mathrm{WT}/(\mathrm{CE}+0.5\times \mathrm{FA}+0.7\times \mathrm{SG}))$ [36]. In Equation (6), the coefficient 0.5 for fly ash and 0.7 for slag in the denominator represents the equivalent carbonation depth coefficients for fly ash and slag, respectively [32]. Equation (6) indicates that as the ambient CO_2_ concentration increases, the carbonation rate accelerates, exacerbating steel reinforcement corrosion caused by carbonation.

Equation (7) calculates the diffusion coefficient of CO_2_, which is related to the pore structure of concrete, environmental humidity, and temperature. In Equation (7), ρ_c_ and ρ_w_ represent the density of cement and water, respectively. *RH* indicates the relative humidity of the surrounding environment; *T_ref_* is the reference temperature (293 K); and *T* stands for the current ambient temperature. β represents the temperature influence coefficient on CO_2_ diffusion, with a value of 4300 [34]. Equation (7) considers the effects of pore structure, environmental relative humidity, and environmental temperature on the diffusion coefficient, respectively. Since climate change causes temperature and CO_2_ concentration to change over time, in order to calculate the carbonation depth, the time-averaged temperature and CO_2_ concentration can be used.

The carbonation depth model requires improvement in the following aspects: First, it assumes that carbonation is solely a carbonation-controlled process. However, at a low relative humidity, the reaction between CO_2_ gas and capillary water governs the carbonation rate. Second, the model assumes similar reaction degrees for different admixtures, which is reasonable in the later stages of hydration but inaccurate for early-stage concrete. The reaction rates follow the order cement > slag > fly ash, with slag and fly ash reacting more slowly than cement. Third, the model does not account for the influence of aggregate pores on CO_2_ diffusion. For lightweight aggregate concrete or concrete with porous aggregates, this omission may lead to significant calculation errors.

**Figure 3:**
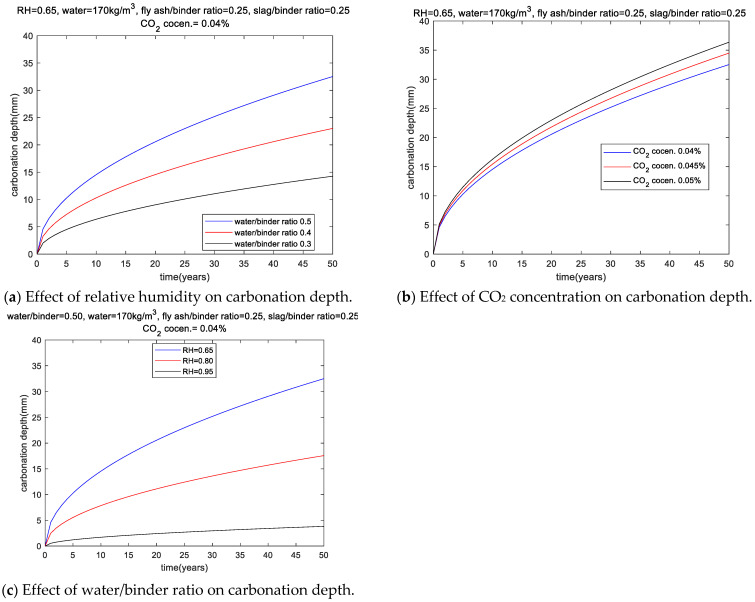
Sensitivity analysis of carbonation depth.

Figure 3a shows the effect of relative humidity on carbonation depth. The water–binder ratio is 0.50, the specific water consumption is 170, and the replacement ratio of slag and fly ash is 0.25. For the three cases of relative humidity equal to 0.65, 0.80, and 0.95, after 50 years of use, the carbonation depth is equal to 32.52 mm, 17.57 mm, and 3.82 mm, respectively. From 0.65 to 0.95, the percentage increase in relative humidity is 46%, and the percentage decrease in carbonation depth is 88%.

Figure 3b shows the effect of CO_2_ concentration on carbonation depth. For the three scenarios of CO_2_ concentration equal to 0.04%, 0.045%, and 0.05%, the carbonation depth is equal to 32.52 mm, 34.49 mm, and 36.36 mm, respectively. From 0.04% to 0.05%, the percentage increase in CO_2_ concentration is 25%, and the percentage increase in carbonation depth is 11%. Compared with relative humidity, the effect of CO_2_ concentration on carbonation depth is not obvious.

Figure 3c shows the effect of water–binder mass ratio on carbonation depth. The relative humidity is 0.65, the specific water consumption is 170, and the replacement ratio of slag and fly ash is 0.25. For the water–binder mass ratios equal to 0.50, 0.40, and 0.30, after 50 years of use, the carbonation depths are 32.52 mm, 23.02 mm, and 14.27 mm, respectively. From 0.50 to 0.30, the water–binder mass ratio decreases by 40%, and the percentage of carbonation depth decreases by 44%.

In summary, the results of Figure 3a–c show that as the relative humidity decreases or the CO_2_ concentration increases, the carbonation depth increases. As the water–binder mass ratio decreases, the carbonation depth also decreases. Relative humidity has a more significant effect on carbonation depth than CO_2_ concentration and the water–binder mass ratio.

The impact of sensitivity analysis results on the optimization design results is as follows: First, when the carbonation depth is low, such as the exposure levels of XC1 and XC2, the carbonation depth is not the dominant factor in the mix ratio, and the compressive strength is the dominant factor in the mix design. Secondly, when the carbonation depth is high, such as the exposure levels of XC3 and XC4, carbonation is the dominant factor in the mix design, and the mass ratio of water–cementitious materials needs to be reduced in order to meet the requirements of carbonation durability. Finally, when the CO_2_ concentration increases, for the exposure levels of XC3 and XC4, the mass ratio of cementitious materials needs to be decreased to meet the requirements of carbonation durability.

Table 1 summarizes the target function and constraints of this study. Overall, the design of low-carbon fly ash–slag concrete is a single-objective optimization problem with constraints. The optimization target is the per-cubic-meter carbon emissions of concrete, with constraints including strength, workability, carbonation durability, and admixture replacement rates. To solve this constrained single-objective optimization problem, the genetic algorithm toolbox in MATLAB was applied to optimize the mass distribution of each binder material within a unit volume of concrete [37]. In the genetic algorithm toolbox of MATLAB, there is a special function (GA) to achieve optimization. This function can consider the optimization goal and optimization constraints, which can be linear constraints or nonlinear constraints. The optimization design goal of this study is to reduce carbon emissions, and the constraints mainly include strength, carbonization durability, and the substitution rate of admixtures.

## 3. Case Studies on Optimal Design

To overcome the shortcomings of previous studies and ensure the carbonation durability of low-carbon slag–fly ash concrete, as shown in Table 2, this study considers three optimization design scenarios. The first scenario does not consider carbonation durability, the second scenario considers carbonation durability at a CO_2_ concentration of 0.04%, and the third scenario considers carbonation durability at a CO_2_ concentration of 0.05%. For each optimization design scenario, four carbonation durability exposure classes are included, resulting in a total of 12 optimized mix designs (3 scenarios × 4 exposure classes = 12 optimized mix designs). For the first design scenario (not considering carbonation durability), the optimized design results corresponding to exposure classes XC1, XC2, XC3, and XC4 are Mix1, Mix2, Mix3, and Mix4, respectively. For the second design scenario (considering carbonation durability at 0.04% CO_2_ concentration), the optimized design results corresponding to exposure classes XC1, XC2, XC3, and XC4 are Mix5, Mix6, Mix7, and Mix8, respectively. For the third design scenario (considering carbonation durability at 0.05% CO_2_ concentration), the optimized design results corresponding to exposure classes XC1, XC2, XC3, and XC4 are Mix9, Mix10, Mix11, and Mix12, respectively. In summary, the comparison between Scenario 2 and Scenario 1 highlights the influence of carbonation durability on the optimal design, while the comparison between Scenario 3 and Scenario 2 demonstrates the effect of increasing CO_2_ concentrations on the optimal design. In the optimization design, the environmental relative humidity for XC1, XC2, XC3, and XC4 is assumed to be 0.95, 0.80, 0.65, and 0.75, respectively [23,27]. The corresponding environmental temperature is the same for all—it is equal to 293 K.

### 3.1. Results of Scenario 1 (Ignore Carbonation Durability)

Table 3 presents the optimized design results for Design Scenario 1. Since carbonation durability was not considered, the optimized design results are identical across all carbonation durability environmental conditions. Table 4 shows the properties of the concrete corresponding to each mix in Scenario 1. For all mixes, the slag replacement rate reached the maximum value of 60%, while the fly ash replacement rate was 15%, not reaching its maximum. This is because, as shown in Equation (1), the carbon emissions per unit mass for cement, slag, and fly ash are 0.93, 0.0265, and 0.0196, respectively. As indicated in Equation (4), the strength coefficients for cement, slag, and fly ash are 21.761, 16.878, and 8.944, respectively. The ratios of carbon emission to strength coefficients are 0.043 (0.93/21.761) for cement, 0.0016 (0.0265/16.878) for slag, and 0.0022 (0.0196/8.944) for fly ash. Compared to other materials, slag has the lowest carbon emission intensity per unit strength. Since the optimization goal of this study is to reduce carbon emissions, the slag replacement rate reached its maximum. Because the carbon emissions per unit strength of both slag and fly ash are significantly lower than those of cement, the total replacement rate of slag and fly ash also reached the upper limit of 0.75.

**Figure 4:**
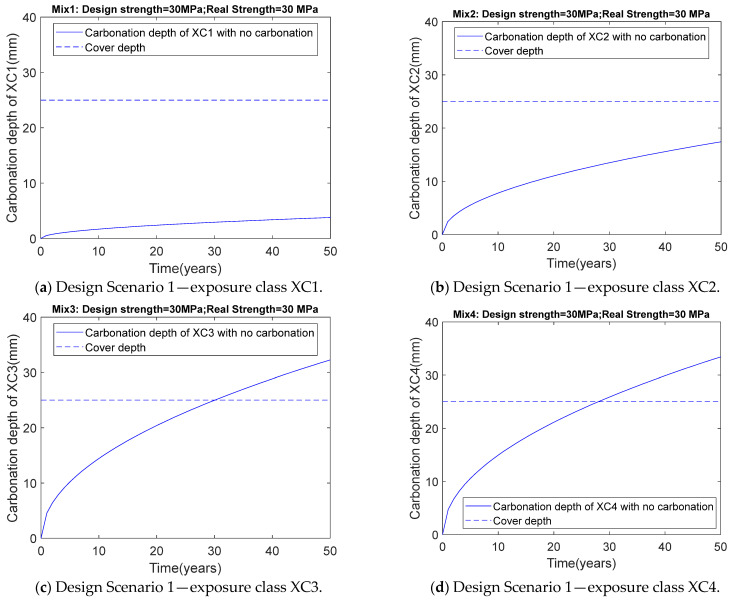
Relationship between carbonation depth and rime for Design Scenario 1.

After obtaining the optimized mix proportions for Scenario 1, the carbonation depth for each mix under different carbonation environmental conditions was calculated using the carbonation depth equation. During the calculation, the temperature and CO_2_ concentration were assumed to be 20 °C (293 K) and 0.04%, respectively. As shown in Table 4 and Figure 4, after 50 years of service, the carbonation depths for XC1 and XC2 are 3.79 mm and 17.43 mm, respectively, both of which are less than the cover thickness, primarily due to the high relative humidity in XC1 and XC2, which limits CO_2_ diffusion. Additionally, as shown in Table 4 and Figure 4, the carbonation depths for XC3 and XC4 are 32.26 mm and 33.42 mm, respectively. From XC3 to XC4, the average relative humidity increases from 0.65 to 0.75, which reduces the diffusion coefficient and carbonation depth. However, for XC4, wet–dry cycles cause microcracks in the concrete, the formation of which accelerates the increase in carbonation depth [34]. The combined effect of these increasing and reducing factors results in a higher carbonation depth for XC4 compared to XC3. Furthermore, the carbonation depths for both XC3 and XC4 exceed the cover thickness of 25 mm, which may lead to steel reinforcement corrosion.

### 3.2. Results of Scenario 2 (Consider Carbonation Durability: 0.04% CO_2_ Concentration)

In the previous section’s design, the constraint of carbonation durability was not considered. This led to carbonation depths exceeding the cover thickness in the XC3 and XC4 carbonation durability exposure classes, which could cause steel reinforcement corrosion and compromise structural integrity. In this section, carbonation durability is included as a constraint in the optimization design. The only difference between Design Scenario 2 in this section and Design Scenario 1 in the previous section is whether carbonation durability is considered. As shown in Table 2, Design Scenario 2 considers carbonation durability, while Design Scenario 1 does not.

After incorporating the carbonation durability constraint, XC1 and XC2 correspond to Mix5 and Mix6, respectively. Mix5 is identical to Mix1, and Mix6 is identical to Mix2. In other words, for the XC1 and XC2 carbonation durability exposure classes, as shown in Table 3, the optimization design results remain unchanged. This is because the high relative humidity in these two scenarios limits CO_2_ diffusion. However, for the XC3 and XC4 carbonation durability exposure classes, different design results emerged. Mix3 and Mix7 correspond to XC3 in Design Scenario 1 and Design Scenario 2, respectively. When carbonation durability is considered, from Mix3 to Mix7, the mass of binder materials increases, as this increase enhances the content of carbonatable substances, reduces porosity, lowers the diffusion coefficient, and decreases carbonation depth. In addition, for exposure class XC4, a similar trend can be found from Mix4 to Mix8 as that from Mix3 to Mix7.

**Figure 5:**
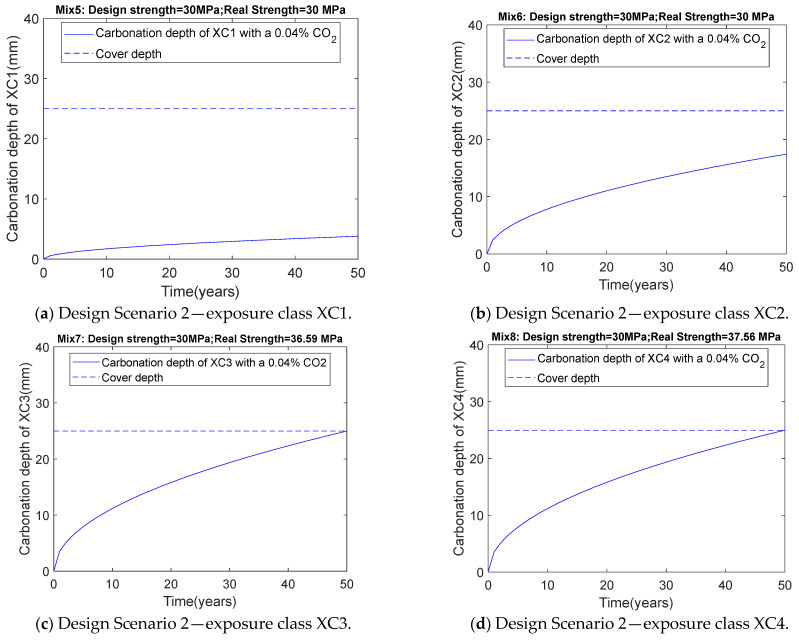
Relationship between carbonation depth and time for Design Scenario 2.

Table 4 and Figure 5 show that after 50 years, the carbonation depth in the XC3 and XC4 exposure classes matches the concrete cover thickness of 25 mm. As indicated in Table 4, for XC3 and XC4, the actual concrete strengths are 36.59 MPa for Mix7 and 37.56 MPa for Mix8, both exceeding the specified design strength of 30 MPa. This indicates that for the XC3 and XC4 carbonation durability exposure classes, carbonation durability is the dominant factor in optimization design, while strength is not the dominant factor. This differs from the results of previous concrete mix design methods. Previous methods, which are similar to Design Scenario 1, assumed compressive strength as the dominant factor in mix design. For the XC3 and XC4 exposure classes, such previous design methods could pose a potential risk of steel corrosion. The design method proposed in this study, which considers the carbonation durability constraint, overcomes the shortcomings of previous concrete design methods and can prevent the corrosion of steel reinforcements due to carbonation.

### 3.3. Results of Scenario 3 (Consider Carbonation Durability: 0.05% CO_2_ Concentration)

In the previous section’s optimization design scenario, the CO_2_ concentration was assumed to be 0.04%. With fossil fuels, deforestation, and industry, the atmospheric CO_2_ concentration is gradually increasing, leading to a greater carbonation depth and heightening the risk of steel reinforcement corrosion. In this section, the impact of increased CO_2_ concentrations on optimization design is considered. As shown in Table 4, for Design Scenario 3, the CO_2_ concentration is assumed to be 0.05%. In other words, Design Scenario 2 considers a typical CO_2_ concentration of 0.04%, while Design Scenario 3 accounts for a CO_2_ concentration of 0.05%. Apart from the CO_2_ concentration, the other constraints in Design Scenario 3 are identical to those in Design Scenario 2.

**Figure 6:**
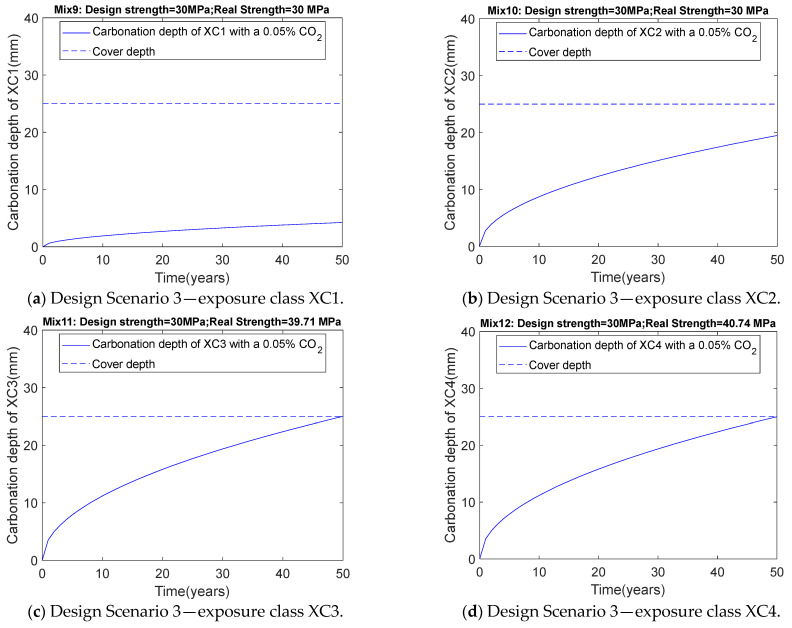
Relationship between carbonation depth and time for Design Scenario 3.

After incorporating the carbonation durability constraint under increasing CO_2_ concentrations, XC1 and XC2 correspond to Mix9 and Mix10, respectively. Mix9 is identical to Mix5, and Mix10 is identical to Mix6. As shown in Table 3, the optimization design results remain unchanged. This is because the high relative humidity in these two scenarios limits CO_2_ diffusion. As indicated in Table 4, when CO_2_ concentration increases, the carbonation depth of the concrete increases. For the XC1 carbonation environment, as the CO_2_ concentration increases from 0.04% in Design Scenario 2 to 0.05% in Design Scenario 3, the carbonation depth rises from 3.79 mm (Mix5) to 4.24 mm (Mix9), while for the XC2 carbonation environment, it increases from 17.43 mm (Mix6) to 19.49 mm (Mix10). Although the carbonation depth increases, as shown in Figure 6a,b, after 50 years of service, the carbonation depth remains below the cover thickness, indicating no risk of steel corrosion. In other words, while CO_2_ concentration rises increase carbonation depth, they do not affect the optimization design results for the XC1 and XC2 exposure classes. This suggests that for the XC1 and XC2 exposure classes, strength (not carbonation durability) is the determining factor in mix design.

As shown in Table 4, for XC3, the measured 28-day compressive strengths with 0.04% and 0.05% CO_2_ concentration considerations are 36.59 MPa (Mix7) and 39.71 MPa (Mix8), respectively. For XC4, the measured 28-day compressive strengths with 0.04% and 0.05% CO_2_ concentrations are 37.56 MPa (Mix11) and 40.74 MPa (Mix11), respectively. In other words, for the XC3 and XC4 carbonation durability exposure classes, when CO_2_ concentration increases are considered, an increase in binder content is required to meet carbonation durability requirements, which also leads to an increase in actual concrete strength. As shown in Figure 6, for the XC3 and XC4 exposure classes, the carbonation depth after 50 years equals the cover thickness. This indicates that for the XC3 and XC4 exposure classes, carbonation durability (not strength) is the determining factor in mix design.

**Figure 7:**
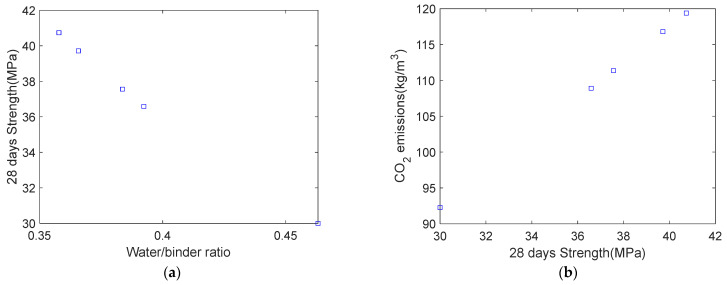
Overall trends in optimization design results. (**a**) Effect of water-to-binder mass ratio on 28-day compressive strength. (**b**) Relationship between carbon emissions and 28-day compressive strength.

In Figure 7a, a clear inverse relationship is observed between the water-to-binder ratio and the 28-day compressive strength [38]. Figure 7b shows that a higher compressive strength leads to greater carbon emissions per unit volume. These patterns reflect practical engineering behavior and qualitatively affirm the soundness of the proposed mix design method [10]. In Figure 7b, the trend of increasing carbon emissions with higher compressive strength aligns with engineering practice. However, this result assumes a constant water consumption per unit volume. If water consumption varies, the trend in Figure 7b may change.

## 4. Discussion

Compared to the mix proportion optimization design methods proposed by previous researchers, the main advantages of this study are as follows.

Firstly, previous optimization approaches primarily emphasized achieving target compressive strength while neglecting the constraint of carbonation durability [10,11,12,13,14,15,16,17,18,19,20,21]. These methods are comparable to Scenario 1 in this study, where it is assumed that satisfying the 28-day compressive strength criterion guarantees adequate carbonation resistance. However, this assumption does not hold true for concretes incorporating fly ash and slag, which may exhibit lower carbonation resistance despite meeting strength requirements. As a result, earlier design strategies may pose a risk of reinforcement corrosion and a potentially reduced structural service life. In contrast, Scenario 2 proposed in this study overcomes these limitations by incorporating both compressive strength and carbonation durability constraints into the optimization framework. For exposure levels of XC1 and XC2, the carbonation depth is less than the thickness of the protective layer. However, for exposure levels of XC3 and XC4, the carbonation depth will exceed the thickness of the protective layer over a period of 50 years, causing the risk of steel corrosion.

Secondly, although previous researchers calculated the durability life considering increasing CO_2_ concentrations, they did not account for the impact of increasing CO_2_ concentrations on concrete mix proportions [39]. In Scenario 3 proposed in this study, the effect of increasing CO_2_ concentrations on carbonation depth was calculated using a carbonation model, and the carbonation durability life under increasing CO_2_ concentrations was included as one of the constraints in the optimization design, clarifying the impact of increasing CO_2_ concentrations on low-carbon concrete design. In other words, while previous researchers identified that increasing CO_2_ concentrations exacerbate carbonation and shorten structural durability life, they did not propose clear solutions. The method proposed in this study not only identifies the problem but also provides a solution.

Thirdly, the strength calculation equation and carbonation depth calculation equation proposed in this study are simple, mechanism-based equations. Despite their simple form, they ensure a certain level of computational accuracy. For the strength calculation equation (Equation (4)), the contributions of cement, slag, and fly ash to strength are considered separately. For the carbonation depth calculation equation (Equations (6) and (7)), the effects of slag and fly ash are accounted for through carbonation durability equivalent coefficients. These mechanism-based equations can be regarded as general equations for predicting strength and carbonation depth, allowing other researchers to adopt similar methods to design the strength and carbonation depth of local low-carbon concrete. Considering variations in materials, curing conditions, and carbonation exposure classes, the equivalent coefficients for strength and carbonation depth derived from experimental results in different regions may differ.

Fourthly, the method proposed in this study can be considered a general design approach. When other researchers apply this method, the following steps are required: (1) A calculation of the per-cubic-meter carbon emissions of concrete based on local life-cycle assessment (LCA) data. Due to differences in LCA data, the results may differ from Equation (1) in this study. (2) A calibration of the strength-equivalent coefficients and carbonation-equivalent coefficients for slag and fly ash based on local experimental results for strength and carbonation. Due to material variations, the coefficients in the strength calculation equation may differ from Equation (4), and those in the carbonation depth calculation equation may differ from Equation (6). (3) After obtaining the equations for compressive strength and carbonation depth, optimized mix proportions can be determined using a genetic algorithm.

## 5. Conclusions

This study proposes an optimized design method for low-carbon fly ash–slag concrete, considering different carbonation durability exposure classes (XC1: fully wet or fully dry; XC2: wet with occasional drying; XC3: moderate relative humidity; and XC4: wet–dry cycles) and CO_2_ concentration increases (CO_2_ concentrations of 0.04% and 0.05%). Through various case studies, the following conclusions are drawn:

1. For Design Scenario 1 (not considering carbonation durability), the actual compressive strength is equal to the design strength. The optimized design results are identical across all carbonation durability environmental conditions. For each mix, the slag replacement rate reached a maximum of 60%, while the fly ash replacement rate was 15%, not reaching its maximum. This is because slag has the lowest carbon emission intensity per unit strength compared to other materials. As the optimization goal of this study is to reduce carbon emissions, the slag replacement rate reached its maximum. Since the carbon emission per unit strength of both slag and fly ash is significantly lower than that of cement, the total replacement rate of fly ash and slag also reached the upper limit of 0.75.
2. For Design Scenario 2 (considering carbonation durability at a 0.04% CO_2_ concentration), when the carbonation durability constraint is included, the optimization design results for XC1 and XC2 remain unchanged compared to Design Scenario 1 (not considering carbonation durability). This is due to the high relative humidity in these two carbonation exposure classes, which limits CO_2_ diffusion. However, for exposure classes XC3 and XC4, different design results emerged, with measured 28-day compressive strength exceeding the design compressive strength. This is because meeting carbonation durability requirements necessitates an increase in binder content, which, in turn, increases the actual compressive strength.
3. For Design Scenario 3 (considering carbonation durability at a 0.05% CO_2_ concentration), for exposure classes XC1 and XC2, although CO_2_ concentration rises increase carbonation depth, they do not affect the optimization design results. This indicates that, similarly to Design Scenarios 1 and 2, strength is the determining factor for mix design in the XC1 and XC2 exposure classes, with the actual compressive strength equaling the design strength. For the XC3 and XC4 exposure classes, when the carbonation durability constraint under increasing CO_2_ concentrations is considered, compared to Design Scenario 2, both the binder content and the actual compressive strength increase to meet carbonation durability requirements. For XC3, the measured 28-day compressive strengths of 0.04% (Design Scenario 2) and 0.05% (Design Scenario 3) CO_2_ concentrations are 36.59 MPa and 39.71 MPa, respectively. For XC4, the measured 28-day compressive strengths are 37.56 MPa (Design Scenario 2) and 40.74 MPa (Design Scenario 3), respectively. For both XC3 and XC4, the carbonation depth after 50 years equals the cover thickness, indicating that carbonation durability, rather than compressive strength, is the determining factor in mix design.
4. Overall, in exposure classes XC1 and XC2, compressive strength serves as the primary governing factor in the optimization process. In these cases, the achieved strength matches the design requirement, and carbonation durability can be reasonably excluded from consideration. Conversely, for the XC3 and XC4 exposure classes, carbonation durability becomes the dominant constraint, resulting in mix designs where the actual compressive strength exceeds the minimum required value to ensure durability. The optimization outcomes also reveal consistent trends, whereby increasing the water-to-binder mass ratio leads to a reduction in compressive strength, while higher 28-day strengths are associated with increased carbon emissions per unit volume of concrete. These findings are consistent with established engineering principles and qualitatively confirm the validity of the proposed optimization method.
5. When the exposure level is XC1 or XC2, carbonation durability can be ignored. However, when the exposure level is XC3 or XC4, carbonation durability must be considered. Engineers adapt this method with local data using the following steps: (1) Calculate the per-cubic-meter carbon emissions of concrete based on local life-cycle assessment (LCA) data. (2) Calibrate the strength-equivalent coefficients and carbonation-equivalent coefficients based on local experimental results for strength and carbonation. (3) After obtaining the equations for compressive strength and carbonation depth, use a genetic algorithm to determine optimized mix proportions. The coefficients in the equations for carbon emissions, strength, and carbonation depth may differ from those used in this study, but the basic steps are similar.
6. The limitations of the proposed method and future improvements are as follows. Although this study proposes a mixed design method for low-carbon fly ash and slag concrete, the following aspects require further improvement: (1) The workability prediction part needs enhancement. Currently, it assumes a constant unit water content for concrete. Future research should consider the effects of maximum coarse aggregate size, sand fineness modulus, binder composition, and environmental conditions (e.g., temperature, humidity, and wind speed) on workability. (2) The carbon emission calculation part needs to account for additional factors, such as carbon emissions from material transportation and concrete production processes. (3) In addition to carbon emissions, other optimization objectives, such as concrete cost, should be further considered in future research. (4) Replacing cement with mineral admixtures reduces the water–binder ratio, potentially requiring water reducers to maintain fluidity. Water reducers are costly, increasing the overall concrete cost. Thus, while mineral admixtures lower carbon emissions, they may raise expenses; this trade-off needs clarification. (5) The optimization in this study focuses on a single objective—reducing carbon emissions. In practice, engineering design often involves multi-objective optimization. If both material costs and carbon emissions are considered, multi-objective optimization design methods can be used, such as the Pareto-optimal design method. When using Pareto optimization, the result is usually a set of compromised solutions (rather than a unique solution). Researchers can select the appropriate ratio on the Pareto frontier according to actual needs.

## Figures and Tables

**Table 1:** Objective function and constraints for optimal design.

Target Function		Minimize Carbon Emissions Per Unit Volume of Concrete
Constraints	Strength constraint	Measured 28-day compressive strength >= design strength
	Water (slump) constraint	Water consumption per unit volume equals 170 kg/m^3^
	Replacement constraint	Fly ash replacement rate is less than 55% Slag replacement rate is less than 60% Total mineral admixture replacement rate is less than 70%
	Carbonation constraint	Carbonation depth <= cover depth

**Table 2:** Carbonation constraint for optimal design.

Carbonation constraint	Scenario 1	Ignore carbonation durability	XC1	Mix1
			XC2	Mix2
			XC3	Mix3
			XC4	Mix4
	Scenario 2	Consider carbonation: 0.04% CO_2_	XC1	Mix5
			XC2	Mix6
			XC3	Mix7
			XC4	Mix8
	Scenario 3	Consider carbonation: 0.05% CO_2_	XC1	Mix9
			XC2	Mix10
			XC3	Mix11
			XC4	Mix12

**Table 3:** Results of mix proportion optimization design.

Scenario	Mix	Water (kg/m^3^)	Cement (kg/m^3^)	Fly Ash (kg/m^3^)	Slag (kg/m^3^)
Scenario 1	Mix1	170.00	91.77	55.06	220.26
	Mix2	170.00	91.77	55.06	220.26
	Mix3	170.00	91.77	55.06	220.26
	Mix4	170.00	91.77	55.06	220.26
Scenario 2	Mix5	170.00	91.77	55.06	220.26
	Mix6	170.00	91.77	55.06	220.26
	Mix7	170.00	108.33	65.00	259.98
	Mix8	170.00	110.77	66.46	265.86
Scenario 3	Mix9	170.00	91.77	55.06	220.26
	Mix10	170.00	91.77	55.06	220.26
	Mix11	170.00	116.19	69.71	278.85
	Mix12	170.00	118.76	71.26	285.02

**Table 4:** Properties of optimized design concrete.

Scenario	Mix	WT /(CE + FA + SG)	FA /(CE + FA + SG)	SG /(CE + FA + SG)	(FA + SG) /(CE + FA + SG)	Carbonation Depth (mm)	Compressive Strength (MPa)	CO_2_ Emission (kg/m^3^)
Scenario 1	Mix1	0.46	0.15	0.60	0.75	3.79	30.00	92.27
	Mix2	0.46	0.15	0.60	0.75	17.43	30.00	92.27
	Mix3	0.46	0.15	0.60	0.75	32.26	30.00	92.27
	Mix4	0.46	0.15	0.60	0.75	33.42	30.00	92.27
Scenario 2	Mix5	0.46	0.15	0.60	0.75	3.79	30.00	92.27
	Mix6	0.46	0.15	0.60	0.75	17.43	30.00	92.27
	Mix7	0.39	0.15	0.60	0.75	25.00	36.59	108.91
	Mix8	0.38	0.15	0.60	0.75	25.00	37.56	111.37
Scenario 3	Mix9	0.46	0.15	0.60	0.75	4.24	30.00	92.27
	Mix10	0.46	0.15	0.60	0.75	19.49	30.00	92.27
	Mix11	0.37	0.15	0.60	0.75	25.00	39.71	116.81
	Mix12	0.36	0.15	0.60	0.75	25.00	40.74	119.40

## Data Availability

The raw data supporting the conclusions of this article will be made available by the authors on request.
